# Tailoring low-dimensional structures of bismuth on monolayer epitaxial graphene

**DOI:** 10.1038/srep11623

**Published:** 2015-06-23

**Authors:** H.-H. Chen, S. H. Su, S.-L. Chang, B.-Y. Cheng, S. W. Chen, H.-Y. Chen, M.-F. Lin, J. C. A. Huang

**Affiliations:** 1 Department of Physics, National Cheng Kung University, Tainan, Taiwan 701, Taiwan; 2 Advanced Optoelectronic Technology Center, National Cheng Kung University, Tainan, Taiwan 701, Taiwan; 3 Taiwan Consortium of Emergent Crystalline Materials, Ministry of Science and Technology, Taipei 106, Taiwan; 4 Department of Electrophysics, National Chiao Tung University, 1001 Ta Hsueh Road, Hsinchu, 30050, Taiwan

## Abstract

To improve graphene-based multifunctional devices at nanoscale, a stepwise and controllable fabrication procedure must be elucidated. Here, a series of structural transition of bismuth (Bi) adatoms, adsorbed on monolayer epitaxial graphene (MEG), is explored at room temperature. Bi adatoms undergo a structural transition from one-dimensional (1D) linear structures to two-dimensional (2D) triangular islands and such 2D growth mode is affected by the corrugated substrate. Upon Bi deposition, a little charge transfer occurs and a characteristic peak can be observed in the tunneling spectrum, reflecting the distinctive electronic structure of the Bi adatoms. When annealed to ~500 K, 2D triangular Bi islands aggregate into Bi nanoclusters (NCs) of uniform size. A well-controlled fabrication method is thus demonstrated. The approaches adopted herein provide perspectives for fabricating and characterizing periodic networks on MEG and related systems, which are useful in realizing graphene-based electronic, energy, sensor and spintronic devices.

Fabrication of low-dimensional structures (LDSs) with novel electronic structures is essential to both fundamental research and device applications. The continuing miniaturization of materials and devices for high-tech industrial applications is a challenge to scientists in various research fields. The main goal is the development of novel LDSs at atomic or nanometer scale with unique physical and chemical properties, resulting from the properties, interactions and processing of building units containing a finite number of atoms. The size distribution and the interactions of LDSs can be controlled to tune fundamental properties including electronic, catalytic and chemical activities. However, a major challenge is the synthesis of well-defined LDSs of uniform atomic/nano size. Therefore a better understanding of the aggregation and reaction sequences is required to provide a controllable, stepwise process for growing LDSs.

Carbon-based LDSs are a promising class of materials for various applications. For example, carbon nanotubes have performed excellently in biosensors and fuel cells. In particular, graphene, single sheet of graphite, is a two-dimensional material resembling massless Dirac fermions^1^, which makes it very different from conventional surfaces and potentially leads to distinct performance of adsorbed species. Additionally, doping graphene with heavy metals can modify the number of carriers and spin-orbit coupling (SOC) can be exploited for novel electronic and spintronic applications^2^,^3^. Graphene also provides a good platform for studying the electrocatalytic effects of carbon materials. Moreover, the integration of graphene in photovoltaic modules, fuel cells, batteries, supercapacitors, and devices for generating hydrogen offers opportunities to overcome the challenges that arise from the increasing global demand for energy^4^,^5^.

Semimetal Bi, being one of the most extensively studied elements, exhibits many extraordinary physical properties, including low carrier density, a long Fermi wavelength, and high carrier mobility^6^. Interestingly, the low-index surfaces of Bi such as Bi (111), Bi (100), and Bi (110) show very different electronic properties from the bulk, owing to SOC splitting at the surface and the breakdown of inversion symmetry^6^. Such Bi surfaces do not react much with O_2_^7^. Additionally, Bi-based LDSs not only form narrow band gap semiconductors due to strong quantum confinement effect^8^,^9^ but are also more active than thin films when their size is restricted to the nanoscale. However, Bi is also used in nanoscale carbon-based materials for the sensing and detection of heavy metals^10^. Bi and bismuth oxide^11^,^12^ have recently been reported as biosensors and electrochemical biosensors^13^. Bismuth oxide can be also used directly as a lithium-ion battery anode^14^ and in fuel cells^15^.

Based on the above discussion, the control of Bi-based LDSs on MEG could be a key precursor for various high-tech applications. In this work, we report the scanning tunneling microscopy (STM) and density functional theory (DFT) studies of Bi adatoms on MEG formed on Si- terminated 4*H*-SiC (0001) substrate. The results reveal the coverage and temperature-dependent structural transition of Bi adatoms at room temperature. With increasing coverages from below 0.01 ML to above 0.03 ML, Bi adatoms experience a structural transition from 1D linear atomic chains to 2D triangular islands and such 2D growth mode is influenced by the corrugated substrate. Upon Bi deposition, a little charge transfer from MEG to Bi adatom and a characteristic peak, corresponding to the p band of Bi, can be observed in tunneling spectrum. Annealing to ~500 K, triangular Bi islands aggregate into uniform Bi NCs. A well-controlled method is comprehensively characterized.

## Results

### STM imagings of MEG and Bi adatoms

**Figure 1:**
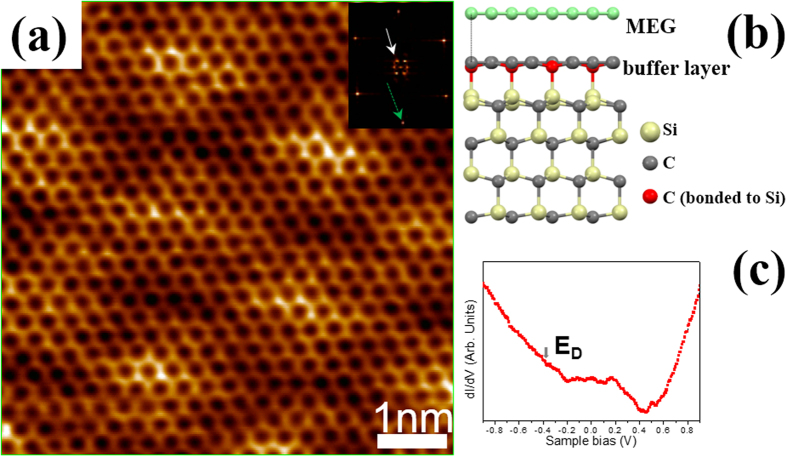
Clean MEG surface. (**a**) Atomically resolved STM image of MEG on 4H-SiC (0001) (6.5 nm × 6.5 nm, *V*_*s*_ = 40 mV, I = 0.15 nA). (**b**) Side view ball-and-stick representations of MEG/4H-SiC (0001). (**c**) dI/dV-V curve obtained on clean surface of MEG.

**Figure 2:**
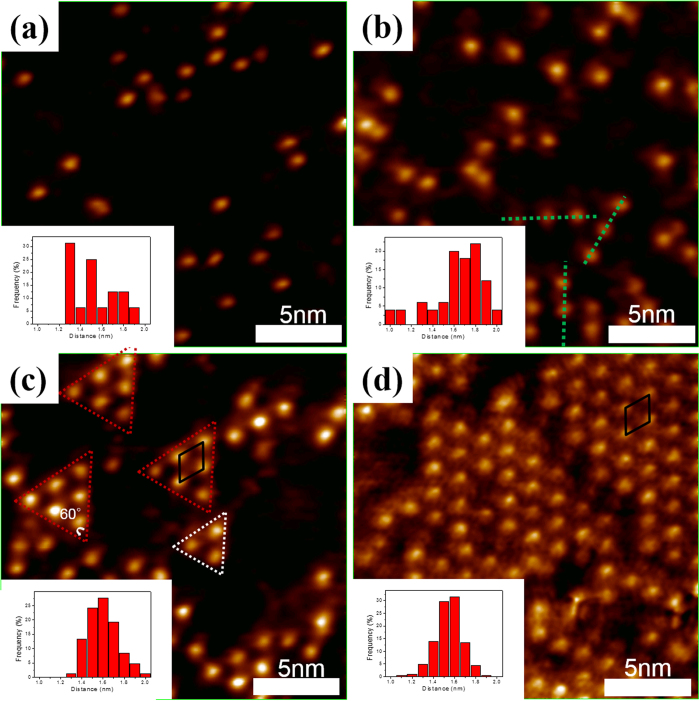
STM images of different Bi coverage on 4H-SiC (0001) surface. (**a**) 0.0013 ML, (**b**) 0.0078 ML, (**c**) 0.039 ML, and (**d**) 0.078 ML. Size of the image is 20 × 20 nm^2^ and the bottom left panel presents the histogram of Bi-Bi interatomic distance. The image conditions are *V*_*s*_ = 0.74 V, I = 0.15 nA for (**b**,**c**) and *V*_*s*_ = 0.85 V, I = 0.15 nA for (**a**) and (**d**).

Fig. 1(a) presents an atomically clean surface of MEG. The image reveals a well-identified honeycomb lattice of the graphene superimposed onto the 6 × 6 superstructure (bright hexagon)^16^,^17^, as well as the corresponding Fast Fourier transform [FFT, inset in Fig. 1(a)]. A white solid arrow points to a 1 × 1-G spot and a green dashed arrow assigns to a 6 × 6-SiC spot^18^,^19^. Fig. 1(b) schematically depicts a cross-section view of the MEG/4H-SiC (0001) structure using ball-and-stick representations. The dI/dV spectrum of the as-grown MEG in Fig. 1(c) exhibits a local minimum ~−0.37 eV, representing the Dirac point of graphene^20^. Although some packets are observed within the range −0.2 eV to 0.4 eV, these are likely deemed to be a result of many-body effects in graphene^21^ or the effect of corrugated substrate. The main feature (Dirac point) still can be identified. Upon the deposition of Bi, protrusions of approximately 0.24 nm in height are observed within different coverage of Bi. The interatomic distance distribution in different coverage is analyzed by means of statistical histogram, as shown in Fig. 2. At 0.0013 ML, dispersed protrusions and local Bi nearest neighbor distances, 1.3 nm and 1.5 nm, are obtained from the histogram [inset in Fig. 2(a)]. The protrusions exhibit linear ordering when the coverage is increased to 0.0078 ML [Fig. 2(b)]. The 1D linear structure appears (green dashed line) in accompany with a Bi-Bi nearest neighbors distance of around 1.8 nm, reflecting a characteristic 1D growth mode at low coverage. Notably, the Bi-Bi distance of 1.8 nm is almost four times the lattice spacing along the [11–20] direction of Bi nanoribbon^22^. At 0.039 ML, 2D triangular islands (indicated by red and white dashed lines) are observed and linear structures are still detected, as presented in Fig. 2(c), revealing the transition from 1D linear to 2D triangular structures. Such 2D triangular islands consist of Bi atoms with equally interatomic distance and form regular triangles, as displayed in Fig. 2(c). In triangular islands, the Bi-Bi distance is about 1.6 nm, which is very close to 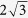 × a = 1.57 nm {a = 4.54 Å, which is the lattice constant along the [11–20] direction of Bi nanoribbon^22^}. The black solid line in Fig. 2(c) delineates a unit cell of Bi adatoms. A large-scale hexagonal array of Bi atoms is formed at 0.078 ML, as presented in Fig. 2(d). The interatomic distance of Bi atoms is maintained at 1.6 nm and the same unit cell is still observed [black solid line in Fig. 2(d)] in the STM images. Therefore, the large-scale 2D hexagonal array is unambiguously identified as coverage-induced 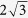 × 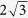 Bi reconstruction on the MEG surface. No ordered structures have been observed in previous literature^23^,^24^,^25^ at such low Bi coverage. The results indicate the existence of some particular interactions between Bi adatoms and MEG. This issue will be addressed in the following discussion.

### Structural analysis of Bi hexagonal array

**Figure 3:**
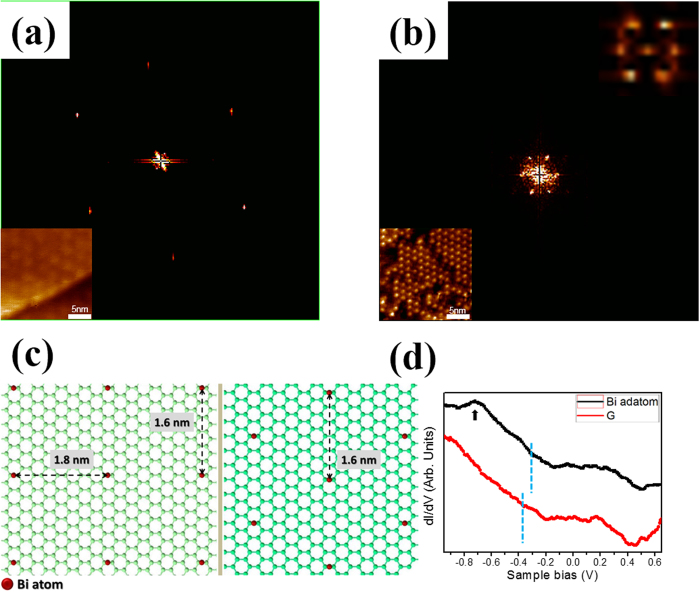
Room-in FFT, atomic structure and electronic structure analysis. Room-in FFT of (**a**) image in bottom left panel (20 × 20 nm^2^ atomically resolved MEG image) and (**b**) Fig. 2d. Top right panel corresponds to zooming-in the FFT of inset in Fig. 1a. (**c**) The theoretically atomic structure of the 1D chain and 2D triangular structures. (**d**) dI/dV spectra of as-grown graphene and Bi adatom in hexagonal array.

The MEG and the Bi hexagonal array are then analyzed by zooming in the FFT of two STM topographic images with same size, as respectively depicted in Fig. 3(a,b). The six outer spots originate from the hexagonal graphene lattice and the six inner spots correspond to the observed 6 × 6-SiC moiré pattern, as shown in Fig. 3(a). The moiré pattern of the MEG is due to the (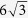 × 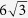) R30° reconstruction of the buffer layer, which occurs as a result of the large difference between lattice parameters of SiC (3.08 Å) and graphene (2.46 Å). Figure 3(b), which zooms in the FFT of Fig. 2(d), shows a similar sixfold symmetry pattern to that of the inner spots in Fig. 3(a). The upper inset, which zooms in the FFT of the inset in Fig. 1(a), shows clearer pattern. That is, the large-scale hexagonal array of Bi adatoms evidences a moiré-like superstructure and such arrangement is strongly affected by the corrugated substrate. As stated above , the interatomic distance between Bi atoms changes from 1.8 nm for the 1D linear chain to 1.6 nm for the 2D triangular island structure, equivalently from four times into 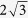 times the lattice spacing of the Bi nanoribbon, indicating a coverage-dependent 1D → 2D growth mode transition in this system. According to the DFT calculations, combined with previous literature^26^,^27^,^28^, Bi adatom is preferentially adsorbed at the bridge (B) site, as shown in Fig. S1 and Table S1. The theoretical arrangements of 1D and 2D structures are then illustrated in Fig. 3(c). Left and right panels reveal the atomic arrangements of Bi adatoms at different coverage, respectively. From the DFT calculations, the interatomic distances between Bi atoms, 1.8 nm and 1.6 nm, coexist at 1D structure dominated configuration while only one characteristic distance of 1.6 nm appears at 2D structure. The DFT results are consistent with the STM observations. This structural transition could be related with coverage-dependent phenomenon and the effect of corrugated substrate. Correspondingly, the electronic properties of Bi adatom in hexagonal array are probed using tunneling spectroscopy, as shown in Fig. 3(d). The dI/dV spectrum of the as-grown MEG exhibits a characteristic minimum at −0.37 eV. This is attributed to Dirac point, indicative of n-type doping by the SiC substrate, while some packets exist around Fermi level (E_F_)^21^. Upon the deposition of Bi adatoms on MEG, the Dirac point shifts to near E_F_, appearing at −0.32 eV [Fig. 3(d)], indicating a little charge transfer (~50 meV) from MEG to Bi adatom. The observed coverage-dependent structural transition and small charge transfer are basically consistent with the STM and synchrotron-based photoemission spectroscopy (PES) measurements^22^. The tunneling spectrum also exhibits one peak at −0.72 eV [black arrow in Fig. 3(d)), which is similar to one of the four characteristic peaks of the Bi (110) nanoribbon, based on rhombohedral indexing^7^,^29^,^30^. As recorded in the literature^30^,^31^, the peak at ~−0.75 eV is mainly attributable to the p-states that are localized at the topmost layer of Bi (110) nanoribbon. Thus, we speculate that the peak in Fig. 3(d) results from the contribution of the p band of 2D Bi hexagonal array. Additionally, the characteristic feature (−0.72 eV) suggests that Bi adatom form a bound state at B site on MEG due likely to the Bi adatom-MEG interaction.

### Temperature effect of Bi adatoms

**Figure 4:**
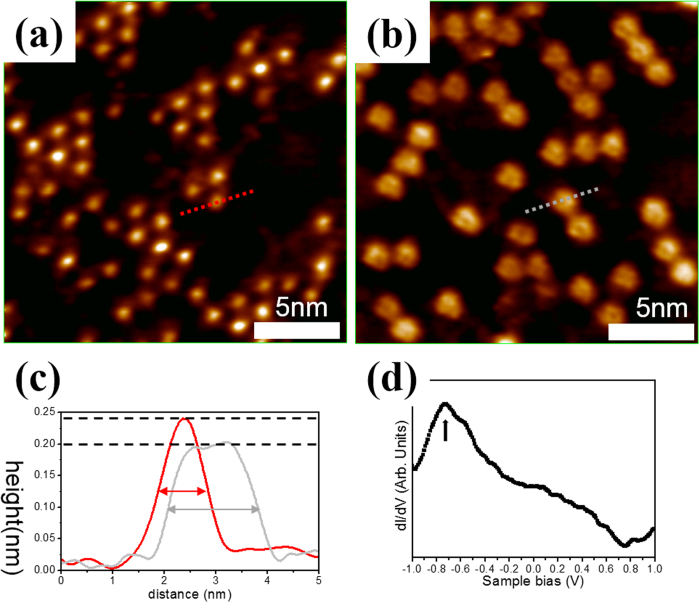
STM images of temperature effect. (**a**) STM image of 0.039 ML Bi coverage. (**b**) Annealed STM image of Fig. 4a. (**c**) Line profile measurements of (**a**) and (**b**). (**d**) dI/dV spectrum of Bi NCs at room temperature. The image size is 20 × 20 nm^2^ and the image conditions are *V*_*s*_ = 0.74 V, I = 0.15 nA for (**a**) and *V*_*s*_ = 0.85 V, I = 0.15 nA for (**b**).

To investigate the effect of temperature, the sample with 0.039 ML of Bi atoms was annealed to an elevated temperature of 500 K. Figure 4(a,b) display the STM images obtained before and after annealing, respectively. After annealing at 500 K for 10 minutes, 1D linear arrangement of Bi NCs, instead of the Bi adatoms in 2D triangular islands, is clearly observed. Interestingly, the Bi NCs are very uniform in size. Within line profile measurements [red and gray dashed lines in Fig. 4(a,b)], the Bi adatoms are ~0.24 nm height and the full width at half maximum (FWHM) ~1 nm in lateral size while Bi NCs are ~0.20 nm height and the FWHM ~2 nm in lateral size, as shown in Fig. 4(c). These results reveal that the Bi NCs are located closer to MEG than are the Bi adatoms. Each Bi NC is likely an aggregation of 3~4 Bi adatoms. Hence, the 2D triangular islands are transformed into the 1D Bi NCs by annealing. Based on the DFT calculations, the atomic model of 1D linear arrangement of Bi NCs are displayed in Fig. S2. Notably, Bi adatoms in NCs remain locating at B site. Moreover, both tri-atoms and quad-atoms configurations of the Bi NCs are possible according to the DFT results. Annealing at a higher elevated temperature ( > 600 K) causes Bi NCs to dissociate into Bi atoms, most of which evaporate from MEG. The spectrum of NCs, as shown in Fig. 4(d), reveal similar characteristic peak located at ~−0.72 eV (black arrow) to that in the dI/dV profile of the Bi adatom in hexagonal array, but with a lager FWHM, indicating stronger feature of the p-state of Bi atoms in NCs. Based on the above, a well-controlled method for forming Bi-based LDSs can be developed. However, further DFT calculations and experimental works to study the effect of corrugated substrate are needed to carry out in the future.

In summary, this work has elucidated the coverage and temperature-dependent structural transition of Bi adatoms adsorbed on MEG that is formed on 4H-SiC (0001) at room temperature. Within STM experiments and DFT calculations, Bi adatoms show a structural transition from 1D linear chains to 2D triangular islands, from four times to 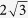 times the lattice spacing of Bi nanoribbon, as coverage increased from below 0.01 ML to above 0.03 ML. Such 2D growth mode is clearly affected by the corrugated substrate. Tunneling spectroscopy measurements reveal a little charge transfer from MEG to Bi adatom and a characteristic peak, corresponding to the p band of Bi, upon deposition of Bi adatoms. After annealing to 500 K, triangular Bi islands aggregate into uniform Bi NCs and reveal stronger feature of the p-state of Bi atoms in NCs. The approaches adopted in this work open up the opportunity to engineer the nucleation and growth of Bi adatoms on MEG. The results also access to a new stepwise route for preparing self-assembled Bi-based low-dimensional structures. Most importantly, it demonstrates a reliable process toward multifunctional hybrid architectures for use in graphene-based devices at room temperature.

## Methods

### Methods of STM experiments and DFT calculations

Experiments were carried out in an ultrahigh-vacuum (UHV) system (JSPM-4500 A/S; JEOL Ltd.) with a base pressure of 2.0 × 10^−8^ Pa. The system consisted of a sample preparation chamber and a variable temperature STM/AFM observation chamber equipped with a cooling tank^32^,^33^. STM experiments were conducted at room temperature and data were collected in a constant current mode. High-resolution topographic STM images were then captured by electrochemically etched 0.35 mm tungsten wires. Before the graphene was imaged, the tip was placed a few μm away from a silicon substrate that had been heated to 1200 °C, indirectly heating and cleaning the tip. Additionally, the tip was also cleaned by applying voltage pulses during STM operation. Moreover, the STM tip is known to influence the size and structure of the atoms. To minimize any artifact from the tip and the STM electronics, the images that are presented in this study were selected only following repeated scans of the same surface region and the evaluation of various similar obtained images. The dI/dV tunneling spectrum was obtained by holding the tip at a fixed distance above the surface and using a standard lock-in technique^33^ at the temperature of liquid nitrogen. A small a.c. modulation voltage was applied to the tip. The calculation results were performed within density functional theory under local density approximation potential (LDA) in the form of Projector augmented-wave method (PAW)^34^,^35^ implemented in the Vienna Ab initio Simulation Package (VASP)^35^,^36^ codes. The buffer layer and monolayer graphene, including 4 bilayer silicon carbide films as the substrate, were constructed. Detailed calculations can be found in our previous work^28^.

### Sample Preparation

The Si-terminated 4H-SiC (0001) single crystal was purchased from Cree, Inc. Si-terminated 4H-SiC (0001) substrate was used and cleaned ultrasonically in acetone and isopropanol before *in situ* degassing at~600 °C in UHV for several hours. The substrate was then annealed under a low Si flux at ~1000 °C to remove the native oxide before it was annealed to higher temperatures in the absence of Si flux. Graphene was subsequently formed by thermal annealing in the temperature range of 1200 °C–1300 °C^37^. The temperature of the sample was measured using a C-type thermocouple and an infrared thermometer (Cyclops 100B, AMETEK Land, Inc.). Bi was deposited on the graphene surface from a Bi rod (with a purity of 99.9999%) by commercial evaporator (Omicrometer EFM 3) at room temperature. The atomic density of the deposited metal was represented in the monolayer (ML) form, corresponding to a Bi packing density of 1.1 × 10^15^ Bi atom/cm^2^ when proceeding along the growth plane of graphene^29^.The growth rate was determined to be approximately 0.0013 ML per minute. Immediately following deposition, the sample was transferred into the STM measurement chamber under UHV conditions.

## Additional Information

**How to cite this article**: Chen, H.-H. *et al*. Tailoring low-dimensional structures of bismuth on monolayer epitaxial graphene. *Sci. Rep.* **5**, 11623; doi: 10.1038/srep11623 (2015).

## Supplementary Material

Supplementary Information
