# Supplementary material for: Tailoring low-dimensional structures of bismuth on monolayer epitaxial graphene
Source: Sci Rep. 2015 Jun 23;5:11623. doi: 10.1038/srep11623 (PMC4477326; doi:10.1038/srep11623)
Supplement: Supplementary Information [file srep11623-s1.doc]

**Tailoring low-dimensional structures of bismuth on monolayer epitaxial graphene**

H.-H. Chen^1^, S. H. Su^1^, S.-L. Chang^4^, B.-Y. Cheng^1^, S. W. Chen^1^, H.-Y. Chen ^1^, M.-F. Lin^1^, J. C. A. Huang^*,1,2,3^

^1^*Department of Physics, National Cheng Kung University, Tainan, Taiwan 701, Taiwan*

^2^*Advanced Optoelectronic Technology Center, National Cheng Kung University, Tainan, Taiwan 701, Taiwan*

^3^*Taiwan Consortium of Emergent Crystalline Materials, Ministry of Science and Technology, Taipei 106, Taiwan*

*^4^Department of Electrophysics, National Chiao Tung University, 1001 Ta Hsueh Road, Hsinchu, Taiwan, 30050*


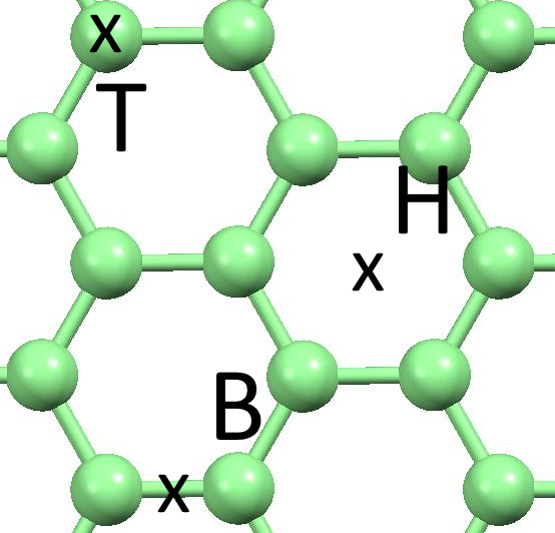


**Fig. S1:** The three adsorption sites considered: hollow (*H*), bridge (*B*), and top (*T*).

**
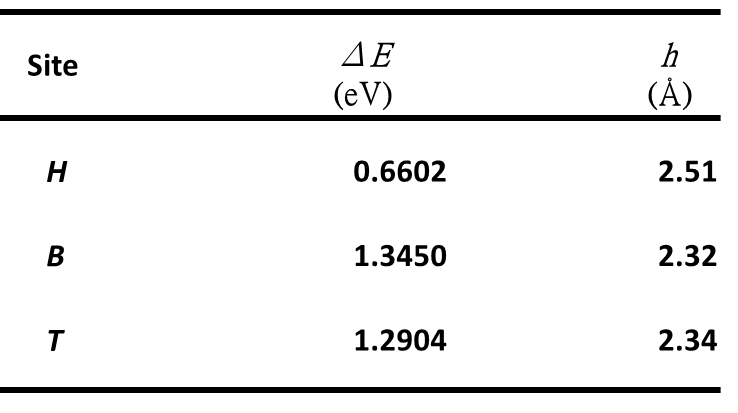
**

**Table S1:** Energetic and structural properties for the *H*, *B*, and *T* sites on graphene. The properties listed are the adsorption energy (*ΔE*) and Bi adatom height (*h*).

**
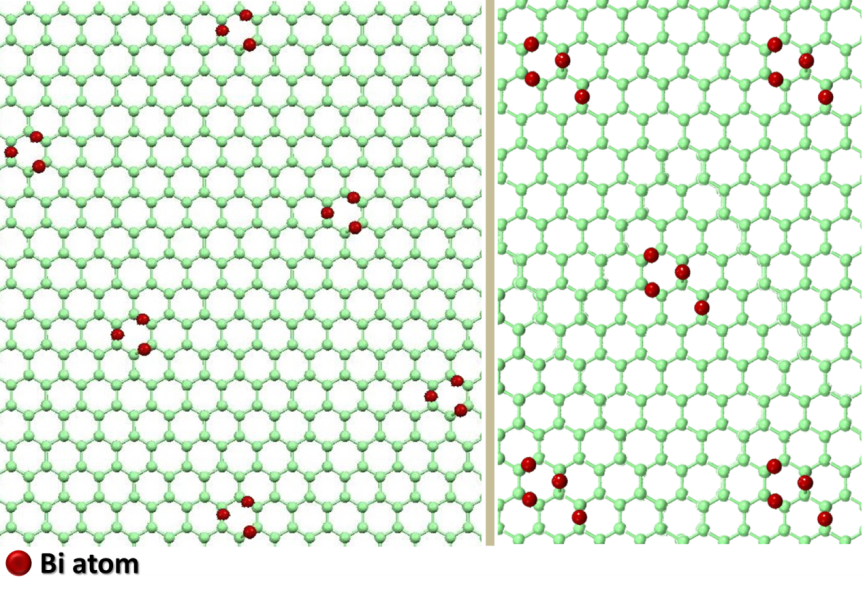
**

**Fig. S2:** The atomic model of 1D linear Bi nanocluster (NC). NCs are consisted of 3 or 4 Bi adatoms, as displayed in left and right panels respectively. The interatomic distance between NCs of 3 Bi adatoms is approximately 2.65 Å.
